# Is Recreational Soccer Effective for Improving $\dot{V}{\text{O}}_{2\;\hbox{max} }$? A Systematic Review and Meta-Analysis

**DOI:** 10.1007/s40279-015-0361-4

**Published:** 2015-07-26

**Authors:** Zoran Milanović, Saša Pantelić, Nedim Čović, Goran Sporiš, Peter Krustrup

**Affiliations:** Faculty of Sport and Physical Education, University of Niš, Niš, Serbia; Faculty of Sport and Physical Education, University of Sarajevo, Sarajevo, Bosnia and Herzegovina; Faculty of Kinesiology, University of Zagreb, Zagreb, Croatia; Sport and Health Sciences, College of Life and Environmental Sciences, University of Exeter, Exeter, UK; Department of Nutrition, Exercise and Sports, Copenhagen Centre for Team Sport and Health, University of Copenhagen, The August Krogh Building, Universitetsparken 13, Copenhagen, 2100 Denmark

## Abstract

**Background:**

Soccer is the most popular sport worldwide, with a long history and currently more than 500 million active participants, of whom 300 million are registered football club members. On the basis of scientific findings showing positive fitness and health effects of recreational soccer, FIFA (Fédération Internationale de Football Association) introduced the slogan “Playing football for 45 min twice a week—best prevention of non-communicable diseases” in 2010.

**Objective:**

The objective of this paper was to perform a systematic review and meta-analysis of the literature to determine the effects of recreational soccer on maximal oxygen uptake ($\dot{V}{\text{O}}_{2\;\hbox{max} }$).

**Methods:**

Six electronic databases (MEDLINE, PubMed, SPORTDiscus, Web of Science, CINAHL and Google Scholar) were searched for original research articles. A manual search was performed to cover the areas of recreational soccer, recreational physical activity, recreational small-sided games and $\dot{V}{\text{O}}_{2\;\hbox{max} }$ using the following key terms, either singly or in combination: recreational small-sided games, recreational football, recreational soccer, street football, street soccer, effect, maximal oxygen uptake, peak oxygen uptake, cardiorespiratory fitness, $\dot{V}{\text{O}}_{2\;\hbox{max} }$. The inclusion criteria were divided into four sections: type of study, type of participants, type of interventions and type of outcome measures. Probabilistic magnitude-based inferences for meta-analysed effects were based on standardised thresholds for small, moderate and large changes (0.2, 0.6 and 1.2, respectively) derived from between-subject standard deviations for baseline fitness.

**Results:**

Seventeen studies met the inclusion criteria and were included in the systematic review and meta-analysis. Mean differences showed that $\dot{V}{\text{O}}_{2\;\hbox{max} }$ increased by 3.51 mL/kg/min (95 % CI 3.07–4.15) over a recreational soccer training programme in comparison with other training models. The meta-analysed effects of recreational soccer on $\dot{V}{\text{O}}_{2\;\hbox{max} }$ compared with the controls of no exercise, continuous running and strength training were most likely largely beneficial [effect size (ES) = 1.46; 95 % confidence interval (CI) 0.91, 2.01; *I*^2^ = 88.35 %], most likely moderately beneficial (ES = 0.68; 95 % CI 0.06, 1.29; *I*^2^ = 69.13 %) and most likely moderately beneficial (ES = 1.08; 95 % CI −0.25, 2.42; *I*^2^ = 71.06 %), respectively. In men and women, the meta-analysed effect was most likely largely beneficial for men (ES = 1.22) and most likely moderately beneficial for women (ES = 0.96) compared with the controls. After 12 weeks of recreational soccer with an intensity of 78–84 % maximal heart rate (HR_max_), healthy untrained men improved their $\dot{V}{\text{O}}_{2\;\hbox{max} }$ by 8–13 %, while untrained elderly participants improved their $\dot{V}{\text{O}}_{2\;\hbox{max} }$ by 15–18 %. Soccer training for 12–70 weeks in healthy women resulted in an improvement in $\dot{V}{\text{O}}_{2\;\hbox{max} }$ of 5–16 %. Significant improvements in $\dot{V}{\text{O}}_{2\;\hbox{max} }$ have been observed in patients with diabetes mellitus, hypertension and prostate cancer.

**Conclusion:**

Recreational soccer produces large improvements in $\dot{V}{\text{O}}_{2\;\hbox{max} }$ compared to strength training and no exercise, regardless of the age, sex and health status of the participants. Furthermore, recreational soccer is better than continuous endurance running, albeit the additional effect is moderate. This kind of physical activity has great potential for enhancing aerobic fitness, and for preventing and treating non-communicable diseases, and is ideal for addressing lack of motivation, a key component in physical (in)activity.

## Key Points

**Table:** 

Recreational soccer is a highly motivating and social activity which produces larger improvements in maximal oxygen uptake ($\dot{V}{\text{O}}_{2\;\hbox{max} }$) than continuous moderate-intensity endurance running, strength training and no-exercise.
$\dot{V}{\text{O}}_{2\;\hbox{max} }$ increases by an average of 3.51 mL/kg/min during a recreational soccer training programme in comparison with other training types.
Recreational soccer is suitable for $\dot{V}{\text{O}}_{2\;\hbox{max} }$ improvement in healthy young and middle-aged people, untrained men and women with mild to moderate hypertension, patients with type 2 diabetes mellitus, untrained elderly people and men with prostate cancer.

## Introduction

Physical inactivity, a major public health problem in both developing and developed countries, is recognised as a global epidemic. The modern sedentary lifestyle contributes to diseases such as hypertension, overweight and hyperglycaemia, which decrease cardiovascular and respiratory functions and reduce functional movement ability [1]. On the other hand, optimal and regular physical exercise is recommended as part of the prevention and treatment of many diseases [2]. Regular physical activity is also effective for maintaining or increasing functional capacity [3], while regular exercise may be a crucial factor in healthy aging [4]. It is well-known that physiological aging causes a decrease of 5–10 % in maximal oxygen uptake ($\dot{V}{\text{O}}_{2\;\hbox{max} }$) per decade [5], and it can impair an independent lifestyle throughout the lifespan [4] if no physical activity is performed. Allender et al. [6] reported that the main barriers to participation in physical activity include high cost, poor access to facilities, and lack of time and motivation.

Soccer is the most popular game in the world (~500 million players worldwide, of whom 300 million are registered football club members) and is associated with positive motivational and social factors, while at the same time contributing to the maintenance of an active lifestyle [7, 8]. It is surprising that up until 2009 all published scientific research articles dealt with elite, sub-elite and amateur soccer players, while recreational soccer and its effect on health-related physical fitness were not represented in the scientific literature, despite the global popularity. However, between 2006 and 2009 a group of Danish researchers conducted several randomised controlled training studies to investigate the effects of recreational soccer on the prevention and treatment of non-communicable diseases across the lifespan. Their global research finding was the prevention of risk factors for non-communicable diseases [8], the maintenance of a physically active lifestyle [7], and the development of positive motivational and social factors [8] in both sexes, regardless of health status. Krustrup et al. [7] concluded that recreational soccer is an effective physical activity for both children and adults, including the elderly, regardless of their physical activity level, health status and lifestyle. Based on the scientific research, FIFA (Fédération Internationale de Football Association) subsequently introduced the slogan “Playing football for 45 min twice a week—best prevention of non-communicable diseases”.

The main characteristic of recreational soccer is varied movement patterns, with ~900 intermittent activity changes per session [7], including high-intensity runs, stop-and-go actions, jumps, sprints, turns and other sport-specific actions such as tackles, dribbles, passes and shots. This kind of physical activity has positive effects on the metabolic and cardiovascular systems as well as on body composition fitness for patients with type 2 diabetes mellitus (T2DM) [3, 9]. As observed in a few recent studies [1, 4], elderly people with no prior soccer experience can use recreational soccer to reboot health fitness, physical capacity and heart function. Some studies provide valid information that playing soccer is effective for treating hypertension in middle-aged men [2, 10, 11] and can increase lean body mass in prostate cancer patients undergoing anti-androgen therapy [12]. The benefits of recreational soccer in untrained people are reflected in improved health profile and physical capacity [13] and enhanced cardiovascular fitness and muscular adaptation performance [14–16]. Krustrup et al. [17] have shown that recreational soccer is as effective as continuous running for $\dot{V}{\text{O}}_{2\;\hbox{max} }$ improvements, assuming a similar number of training hours. Further, in contrast to comparable running groups, $\dot{V}{\text{O}}_{2\;\hbox{max} }$ continues to increase after 4 weeks, indicating that soccer maintains the stimuli for cardiovascular and respiratory adaptations throughout the entire training period [17].

It is therefore not surprising that recent meta-analyses have confirmed that high-intensity interval training (HIIT) [18], sprint interval training [19, 20] and continuous endurance running [21–23] provide adequate stimuli for improving $\dot{V}{\text{O}}_{2\;\hbox{max} }$ in healthy people. Also, there have been several meta-analysis papers confirming that high-intensity training, continuous-exercise endurance running and strength training help to improve $\dot{V}{\text{O}}_{2\;\hbox{max} }$ in patient populations with lifestyle-induced cardiometabolic disease [24], hypertension [25], T2DM [26] and obesity [27]. Based on what is known about the potential benefits of recreational soccer, Krustrup et al. [17] published a topical review aimed at describing the effects of regular recreational soccer training on cardiorespiratory fitness, metabolic fitness and musculo-skeletal fitness. To the best of the authors’ knowledge, there has been no systematic review and meta-analysis to determine the effect of recreational soccer on $\dot{V}{\text{O}}_{2\;\hbox{max} }$ regardless of age, sex and training status in both healthy and patient populations. Furthermore, no meta-analysis has compared the effect of recreational soccer with more conventional and previously confirmed training models such as running or strength training. Consequently, the purpose of the present paper was to (1) systematically review the results of the published scientific papers concerning the effects of recreational soccer on physical fitness; (2) use meta-analysis to provide estimates of the effect of recreational soccer on $\dot{V}{\text{O}}_{2\;\hbox{max} }$ in men and women; and (3) assess the efficacy of recreational soccer in comparison with a no-exercise (control) group, endurance running and strength training. We hypothesised that the combined use of a large number of different training components in recreational soccer produces significant improvements in $\dot{V}{\text{O}}_{2\;\hbox{max} }$.

## Methods

### Search Strategy and Study Selection

Electronic database searches were performed in MEDLINE, PubMed, SPORTDiscus, Web of Science, CINAHL and Google Scholar using all available records up to 10 October 2014. Google Scholar alerts were set up in January 2012 to identify potential papers with the following key terms: recreational soccer, recreational football and street soccer. Apart from the Google Scholar alerts, a manual search was performed covering the areas of recreational soccer, recreational physical activity, recreational small-sided games and $\dot{V}{\text{O}}_{2\;\hbox{max} }$ using the following key terms, either singly or in combination: recreational small-sided games, recreational football, recreational soccer, street soccer, street football, effect, maximal oxygen uptake, peak oxygen uptake, cardiorespiratory fitness, $\dot{V}{\text{O}}_{2\;\hbox{max} }$. Reference lists from retrieved manuscripts were also examined for any other potentially eligible papers.

**Fig. 1:**
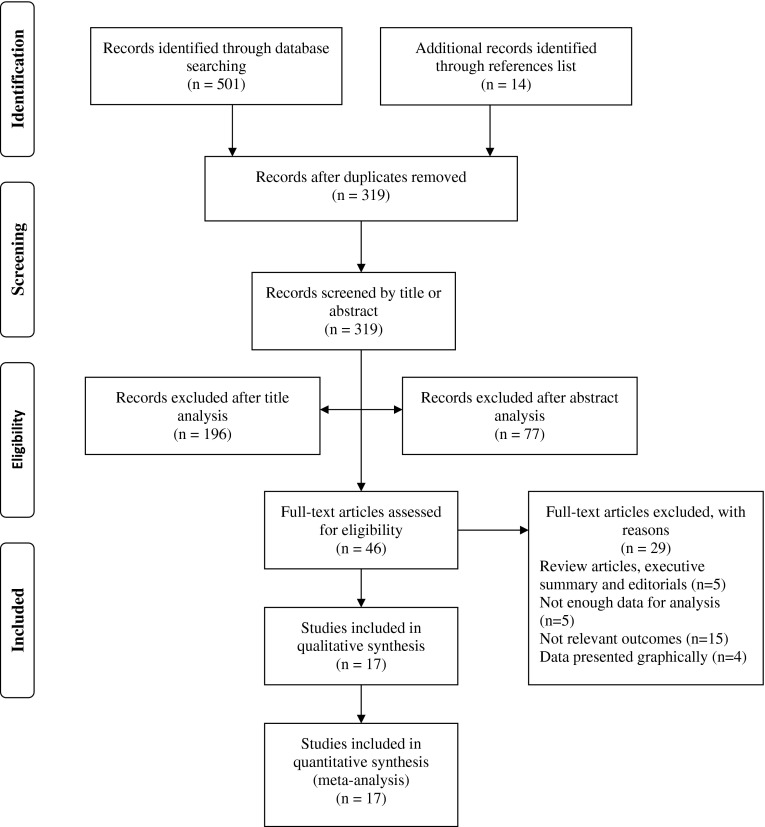
Flow chart diagram of the study selection

The literature search, identification, screening, quality assessment and data extraction were conducted independently by two reviewers (ZM and GS). To identify relevant papers, all titles were initially screened by the reviewers during the electronic searches to exclude manuscripts that were beyond the scope of this meta-analysis. The initial screening process identified 501 potentially eligible papers. Papers that were clearly not relevant were removed from the database list before abstracts were assessed using predetermined inclusion and exclusion criteria. The process of the study selection is shown in Fig. 1. The full texts of the remaining papers that met the inclusion criteria were included in the ongoing procedure and reviewed by the two reviewers to reach a final decision on inclusion in the meta-analysis. Disagreements between the reviewers were resolved by consensus or arbitration through a third reviewer (NČ). The full papers, including reviews, were then retrieved and, if not available, the corresponding author was contacted by mail. This systematic review and meta-analysis was undertaken in accordance with the Preferred Reporting Items for Systematic Reviews and Meta-Analyses (PRISMA) Statement [28].

### Inclusion Criteria

#### Type of Study

Longitudinal design-evaluating interventions, randomised controlled trials and matched controlled trials written in English were reviewed, while non-randomised, uncontrolled and cross-section studies were excluded from further analysis. No publication data or publication status restrictions were imposed.

#### Type of Participants

Sedentary/untrained, recreational non-athletes, including patients, of either sex and of any age and health status were included. No inclusion criteria for the participants’ baseline fitness level were applied.

#### Type of Interventions

Training programmes had to last at least 2 weeks, with participants allocated to a recreational soccer group, a continuous endurance running group, a strength training group or a no-exercise (control) group. Studies incorporating diet were included if the diet was used by all participants in all groups. Number of training sessions per week and training intensity were not used as inclusion criteria.

#### Type of Outcome Measures

The primary outcome measure for the meta-analysis was $\dot{V}{\text{O}}_{2\;\hbox{max} }$.

### Exclusion Criteria

The exclusion criteria were as follows: (1) non-randomised studies; (2) studies written in languages other than English; (3) studies without a control group or without two exercise groups; (4) duplicate publications; (5) studies with training programmes lasting less than 2 weeks; and (6) studies where the results were graphically presented without the relevant data necessary for meta-analysis.

### Data Extraction

The Cochrane Consumers and Communication Review Group’s data extraction standardised protocol was used to extract (1) study characteristics, including author(s), title and year of publication; (2) participant information such as sample size, age, health status and sex; (3) description of the training intervention, including types of exercise, intensity, duration and frequency; and (4) study outcomes, including health-related physical fitness components for systematic review and $\dot{V}{\text{O}}_{2\;\hbox{max} }$ values in mL/kg/min for meta-analysis (Table 1). When needed, pre- and post-$\dot{V}{\text{O}}_{2\;\hbox{max} }$ values were converted from absolute (L/min) to relative (mL/kg/min) values. In most of the studies, mean and standard deviation (SD) pre and post values were reported, while correlation was not reported. Accordingly, in these instances the correlation value was set at 0.5, as used previously by Bacon et al. [18]. Data extraction was undertaken by ZM, while GS checked the extracted data for accuracy and completeness. Disagreements were resolved by consensus or by NČ. The reviewers were not blinded to authors, institutions or manuscript journals.

**Table 1:** Summary of characteristics of all studies meeting the inclusion criteria

Study name	Population, age (*n*)	Comparison group	Δ $\dot{V}{\text{O}}_{2\;\hbox{max} }$ (%)	Duration (weeks)	Training programme: intensity, frequency, duration of session	Outcomes and results
Krustrup et al. [13]	Healthy untrained Danish men	FG (*n* = 13)	12.63	12	FG: 82 % HR_max_	FG: $\dot{V}{\text{O}}_{2\;\hbox{max} }$ (13 % ↑*), SBP (8 mmHg ↓*), DBP (5 mmHg ↓*), fat mass (2.7 kg ↓*), LBM (1.7 kg ↑*), leg bone mass (41 g ↑*), LDL (0.4 mmol/L ↓*) RG: $\dot{V}{\text{O}}_{2\;\hbox{max} }$ (8 % ↑*), SBP (8 mmHg ↓*), DBP (5 mmHg ↓*), fat mass (1.8 kg ↓*) CG: no changes
	20–43 years (*n* = 36)	RG (*n* = 12)	7.38		RG: 82 % HR_max_	
		CG (*n* = 11)	−0.77		CG: maintained their lifestyle	
Randers et al. [14]	Healthy untrained men	FG (*n* = 10)	8.06	12	FG—first 12-week period: 81 % HR_max_; 2.4 (1.8–2.9) times/week; 60 min	FG: fat mass (3.2 kg ↓*), SBP (8 mmHg ↓*), $\dot{V}{\text{O}}_{2\;\hbox{max} }$ (8 % ↑*), Yo–Yo IE2 test (49 % ↑*), ITT (14 % ↑*), RHR (7 ± 2 bpm ↓*), QMM (11 % ↑*), fibre area (10 % ↑*), leg bone mass (3.5 % ↑*), bone density (2 % ↑*), 30 m sprint (1.3–3 % ↑*), BL (27–72 % ↓*) CG: no changes
	20–43 years (*n* = 17)	CG (*n* = 7)	−2.27	52 follow-up	FG—second 52-week period: 82 % HR_max_; 1.3 (0.9–1.6) times/week; 60 min CG: maintained their lifestyle	
	Dropouts (*n* = 5)					
Andersen et al. [10]	Untrained men with mild to moderate hypertension	FG (*n* = 13)	7.69	12	FG: 83 % HR_max_; 1.7 ± 0.2 times/week; 60 min	FG: $\dot{V}{\text{O}}_{2\;\hbox{max} }$ (8 ± 2 % ↑*), SBP (12 ± 3 mmHg ↓*), DBP (7 ± 1 mmHg ↓*), MAP (9 ± 2 mmHg ↓*), fat mass (1.7 ± 0.6 kg ↓*), TFP (5 ± 2 % ↓*) CG: no changes
	31–54 years (*n* = 22)	CG (*n* = 9)	−3.58		CG: advised by cardiologist	
	Dropouts (*n* = 3)					
Knoepfli‐Lenzin et al. [11]	Untrained men with mild hypertension	FG (*n* = 15)	8.68	12	FG: 79.9 ± 4.5 % HR_max_; 2.4 ± 0.2 times/week; 59 ± 2 min	FG: $\dot{V}{\text{O}}_{2\;\hbox{max} }$ (9 % ↑*), RHR (10.3 % ↓*), MSV (13.1 % ↑*), SBP (7.5 % ↓*), DBP (10.3 % ↓*), MAP (10 ± 7 mmHg ↓*), fat mass (2.0 ± 1.5 kg ↓*), TFP (2 % ↓*), Total C (5.2 % ↓*) RG: $\dot{V}{\text{O}}_{2\;\hbox{max} }$ (12 % ↑*), RHR (12.9 % ↓*), MSV (10 % ↑*), SBP (5.9 % ↓*), MAP (6 ± 8 mmHg ↓*), DBP (6.9 % ↓*), fat mass (1.6 ± 1.5 kg ↓*), TFP (no changes ↓*), MSV (10.1 % ↑*), Total C (no changes ↓*) CG: no changes
	20–45 years (*n* = 47)	RG (*n* = 15)	12.17		RG: 79.4 ± 1.3 % HR_max_; 2.5 ± 0.3 times/week; 58 ± 3 min	
	Dropouts (*n* = 10)	CG (*n* = 17)	0.92		CG: maintained their lifestyle	
Krustrup et al. [16]	Healthy untrained men	FG (*n* = 12)	12.88	12	FG: 82 ± 2 % HR_max_; 2.3 times/week; 60 min	FG: MMFA (15 % ↑*), QMM (9 % ↑*), CPF (22 % ↑*), 30 m (0.11 ± 0.02 ↑*), MIHS (11 % ↑*), Yo–Yo IE2 (37 % ↑*), $\dot{V}{\text{O}}_{2\;\hbox{max} }$ (13 % ↑*), BM (1.1 ± 0.2 kg ↓*), WRHR (13–22 bpm ↓*) RG: CPF (16 % ↑*), Yo–Yo IE2 (36 % ↑*), $\dot{V}{\text{O}}_{2\;\hbox{max} }$ (6 % ↑*), BM (1.0 ± 0.3 kg ↓*), WRHR (14–23 bpm ↓*) CG: no changes
	20–43 years (*n* = 38)	RG (*n* = 10)	5.85		RG: 82 ± 1 % HR_max_; 2.5 times/week; 60 min	
	Dropouts (*n* = 6)	CG (*n* = 10)	−0.26		CG: maintained their lifestyle	
Randers et al. [15]	Homeless men	FG (*n* = 22)	10.63	12	FG: 82 ± 4 % HR_max_; 2.8 ± 0.8 times/week; 60 min	FG: $\dot{V}{\text{O}}_{2\;\hbox{max} }$ (10 % ↑*), fat mass (1.6 kg ↓*), TFP (1.9 % ↓*), LDL (6 % ↓*), HDL:LDL (0.6 ↑*), Yo–Yo IE1 (45 % ↑*) CG: no changes
	27–47 years (*n* = 32)	CG (*n* = 10)	−0.89		CG: maintained their lifestyle	
	Dropouts (*n* = 23)					
Schmidt et al. [1]	Untrained men	FG (*n* = 9)	8.73	52	FG: 1.7 ± 0.3 times/week	FG: $\dot{V}{\text{O}}_{2\;\hbox{max} }$ (18 % ↑*), RHR (6–8 bpm ↓*) CG: no changes
	65–75 years (*n* = 26)	STG (*n* = 9)	0.00		STG: 3–4 sets of 12, 10, 8 RM, 1.9 ± 0.2 times/week	
	Dropouts (*n* = 1)	CG (*n* = 8)	0.00		CG: maintained their lifestyle	
Andersen et al. [2]	Untrained hypertensive men	FG (*n* = 22)	7.72	26	FG: 1.7 ± 0.1 times/week; 60 min	FG: SBP (12 mmHg ↓*), DBP (8 mmHg ↓*), RHR (8 bpm ↓*) CG: SBP (6 mmHg ↓*), DBP (4 mmHg ↓*), RHR (3 bpm ↓*)
	31–54 years (*n* = 31)	CG (*n* = 11)	−1.88		CG: advised by cardiologist	
	Dropouts (*n* = 0)					
Andersen et al. [4]	Untrained men	FG (*n* = 9)	13.48	16	FG: 84 ± 1 % HR_max_; 1.6 ± 0.1 times/week; 60 min	FG: $\dot{V}{\text{O}}_{2\;\hbox{max} }$ (15 % ↑*), Yo–Yo IE1 (43 % ↑*), HRdw (12 % ↓*) STG: HRdw (10 % ↓*) CG: no changes
	63–74 years (*n* = 26)	STG (*n* = 9)	2.67		STG: 61 ± 3 % HR_max_; 1.5 ± 0.1 times/week; 5 exercises, 12 weeks 3 sets, 4 weeks 4 sets; 20 to 8 RM	
	Dropouts (*n* = 1)	CG (*n* = 8)	−2.27			
Andersen et al. [3]	Men with type 2 diabetes	FG (*n* = 12)	11.80	24	FG: 83 ± 2 % HR_max_; 1.5 ± 0.9 times/week; 60 min	FG: $\dot{V}{\text{O}}_{{2\;{\text{peak}}}}$ (11 % ↑*), fat mass (5.7 % ↓*) CG: leg lean mass (3.5 % ↓*), leg bone mass (1.6 % ↓*)
	49.8 ± 1.7 years (*n* = 21)	CG (*n* = 9)	0.73		CG: maintained their lifestyle	
	Dropout (*n* = 3)					
Uth et al. [12]	Men with prostate cancer	FG (*n* = 29)	5.51	12	FG: 84.6 ± 3.9 HR_max_; 1.7 ± 0.1 times/week; 60 min	FG: LBM (2.7 % ↑*), knee extensor 1 RM (8.9 % ↑*), fat mass (2.8 % ↓*), $\dot{V}{\text{O}}_{2\;\hbox{max} }$ (5.3 % ↑*) CG: no changes
	43–74 years (*n* = 57)	CG (*n* = 28)	1.89		CG: under regular treatment	
	Dropouts (*n* = 8)					
Krustrup et al. [34]	Healthy untrained premenopausal women	FG (*n* = 21)	15.29	16	FG: 83 % HR_max_; 1.8 times/week; 60 min	FG: MAP (5 ± 1 mmHg ↓*), SBP (7 ± 2 mmHg ↓*), DBP (4 ± 1 mmHg ↓*), RHR (5 ± 1 bpm ↓*), $\dot{V}{\text{O}}_{2\;\hbox{max} }$ (15 % ↑*), fat mass (1.4 ± 0.3 kg ↓*), LBM (1.4 ± 0.3 kg ↑*) RG: MAP (3 ± 1 mmHg ↓*), SBP (6 ± 2 mmHg ↓*), RHR (5 ± 1 bpm ↓*), $\dot{V}{\text{O}}_{2\;\hbox{max} }$ (10 % ↑*), fat mass (1.1 ± 0.3 kg ↓*), LBM (1.3 ± 0.3 kg ↑*) CG: no changes
	19–47 years (*n* = 53)	RG (*n* = 18)	10.14		RG: 82 % HR_max_; 1.85 times/week; 60 min	
	Dropouts (*n* = 12)	CG (*n* = 14)	2.01		CG: maintained their lifestyle	
Andersen et al. [35]	Healthy untrained premenopausal women	FG (*n* = 19)	15.38	16	FG: 82 % HR_max_; 1.8 times/week; 60 min	FG: $\dot{V}{\text{O}}_{2\;\hbox{max} }$ (16 % ↑*), MAP (5 mmHg ↓*), RHR (5 bpm ↓*) RG: $\dot{V}{\text{O}}_{2\;\hbox{max} }$ (10 % ↑*), MAP (3 mmHg ↓*), RHR (5 bpm ↓*) CG: no changes
	36.5 ± 8.2 years (*n* = 47)	RG (*n* = 18)	10.14		RG: 82 % HR_max_; 1.9 times/week; 60 min	
	Dropouts (*n* = 0)	CG (*n* = 10)	0.00		CG: maintained their lifestyle	
Krustrup et al. [36]	Healthy untrained women	FG (*n* = 7)	13.99	70	FG: 81 ± 1 % HR_max_; 1.78 times/week; 60 min	FG: BMD (2.3 ± 0.4 % ↑*), LBM (1.0 kg ↑*), MVC (12 % ↑*), RFD (35 % ↑*), MIHS (23 % ↑*), Slt (27 % ↓*), Sld (42 % ↓*), PBll (42 % ↓*), PBrl (53 % ↓*), $\dot{V}{\text{O}}_{2\;\hbox{max} }$ (14 % ↑*), Yo–Yo IE2 (24 % ↑*), ITT (26 % ↑*) RG: Slt (14 % ↓*), Sld (29 % ↓*), PBll (38 % ↓*), PBrl (42 % ↓*), $\dot{V}{\text{O}}_{2\;\hbox{max} }$ (13 % ↑*), Yo–Yo IE2 (29 % ↑*), ITT (27 % ↑*) CG: no changes
	19–47 years (*n* = 22)	RG (*n* = 8)	13.04		RG: 82 ± 1 % HR_max_; 1.74 times/week; 60 min	
	Dropouts (*n* = 6)	CG (*n* = 7)	1.36		CG: maintained their lifestyle	
Barene et al. [37]	Healthy women hospital employees	FG (*n* = 31)	4.57	12	FG: 78.3 ± 4.4 % HR_max_; 2.4 ± 0.5 times/week; 60 min	FG: $\dot{V}{\text{O}}_{{2\;{\text{peak}}}}$ (5 % ↑*), HR_submax_ at 100 W (1.1 % ↓*), fat mass (1 kg ↓*), HR_mean_ (6.7 bpm ↓*), BFper (1.1 % ↓*), BMC (32.5 g ↑*), POAE (7.6 W ↑*) ZG: $\dot{V}{\text{O}}_{{2\;{\text{peak}}}}$ (5 % ↑*), fat mass (0.6 kg ↓*), POAE (13.4 W ↑*) CG: no changes
	25–65 years (*n* = 118)	ZG (*n* = 30)	4.72		ZG: 75.3 ± 7.1 % HR_max_; 2.3 ± 0.3 times/week; 60 min	
	Dropouts (*n* = 23)	CG (*n* = 34)	0.00		CG: maintained their lifestyle	
Barene et al. [33]	Healthy women hospital employees	FG (*n* = 37)	3.35	40	FG: 78.6 ± 3.2 % HR_max_; 2.4 ± 0.5 times/week; 12 weeks, 1.2 ± 0.2 times/week; 28 weeks, 60 min	FG: lower-limb BMD (0.05 g/cm^2^ ↑*), fat mass (1.2 kg ↓*), total BMD (0.8 % ↑*), total BMC (39.3 g ↑*) ZG: $\dot{V}{\text{O}}_{{2\;{\text{peak}}}}$ (2.2 mmol/L ↑*), power output (12 W ↑*), fat mass (1.3 kg ↓*), BMI (0.7 kg/m^2^ ↓*), body weight (2.1 kg ↓*) CG: no changes
	25–65 years (*n* = 118)	ZG (*n* = 35)	4.09		ZG: 74.9 ± 7.2 % HR_max_; 2.3 ± 0.3 times/week for 12 weeks; 1.5 ± 0.2 times/week for 28 weeks; 60 min	
	Dropouts (*n* = 41)	CG (*n* = 35)	0.00		CG: maintained their lifestyle	
Sousa et al. [9]	Type 2 diabetics	FG (*n* = 22)	9.61	12	FG: 3 vs. 3, 7 vs. 7, for 40 min 3 times/week; +diet	FG: $\dot{V}{\text{O}}_{2\;\hbox{max} }$ (10 ± 4 % ↑*), blood triglycerides (0.4 ± 0.1 mmol/L ↓*), total cholesterol (0.6 ± 0.2 mmol/L ↓*), fat mass (3.4 ± 0.4 kg ↓*), fasting glucose (1.1 ± 0.2 mmol/L ↓*), BW (3.7 ± 0.6 kg ↓*), WC (5.4 ± 0.2 cm ↓*) CG: $\dot{V}{\text{O}}_{2\;\hbox{max} }$ (3 ± 4 % ↓*), fat mass (3.7 ± 0.4 kg ↓*), BW (4.7 ± 0.7 kg ↓*), WC (6.2 ± 0.1 cm ↓*)
	48–68 years: men (*n* = 17); women (*n* = 27)	CG (*n* = 22)	−3.21		CG: diet	
	Dropouts (*n* = 10)					

*Δ* $\dot{V}{\text{O}}_{2\;\hbox{max} }$ change in $\dot{V}{\text{O}}_{2\;\hbox{max} }$, *BFper* body fat percentage, *BL* blood lactate, *BM* body mass, *BMI* body mass index, *BMC* bone mineral content, *BMD* bone mineral density, *bpm* beats per min, *BW* body weight, *CG* control group, *CPF* capillaries per fibre, *DBP* diastolic blood pressure, *FG* football group, *HDL* high-density lipoprotein, *HDL:LDL* cholesterol ratio, *HRdw* heart rate during walk, *HR*_*max*_ maximal heart rate, *HR*_*mean*_ mean heart rate, *HR*_*submax*_ submaximal heart rate, *ITT* incremental treadmill test, *LBM* lean body mass, *LDL* low-density lipoproteins, *MAP* mean arterial pressure, *MIHS* maximal isometric hamstring strength, *MMFA* mean muscle fibre area, *MSV* maximal stroke volume, *MVC* maximal isometric quadriceps contraction strength, *PBll* postural balance left leg, *PBrl* postural balance right leg, *POAE* power output at exhaustion, *QMM* quadriceps muscle mass, *RFD* contractile rate of force development, *RG* running group, *RHR* resting heart rate, *RM* repetition maximum, *SBP* systolic blood pressure, *Sld* distance of sudden trunk loading, *Slt* time of sudden trunk loading, *STG* strength group, *TFP* total fat percentage, *Total* *C* total cholesterol, $\dot{V}{\text{O}}_{2\;\hbox{max} }$ maximal oxygen uptake, $\dot{V}{\text{O}}_{{2\;{\text{peak}}}}$ peak oxygen uptake, *WC* waist circumference, *WRHR* walking and running heart rate, *Yo–Yo IE1* Yo–Yo intermittent endurance test level 1, *Yo–Yo IE2* intermittent endurance test level 2, *ZG* zumba group, ↑* significant increases, ↓* significant decreases

### Assessment of Risk of Bias

Risk of bias was evaluated according to the PRISMA recommendation [29]. Two independent reviewers assessed the risk of bias. Agreement between the two reviewers was assessed using *k* statistics for full-text screening and rating of relevance and risk of bias. In the event of disagreement about the risk of bias, the third reviewer checked the data and took the final decision on it. The *k* agreement rate between reviewers was *k* = 0.94.

### Statistical Analysis

The standardised mean differences and 95 % confidence intervals (CIs) were calculated for the included studies. The *I*^2^ measure of inconsistency was used to examine between-study variability, with values greater than 50 % considered indicative of high heterogeneity [30]. This statistic, expressed as a percentage between 0 and 100 %, can be interpreted as the percentage of heterogeneity in the system or, basically, the amount of total variation accounted for by the between-studies variance [31]. Publication bias was assessed by examining asymmetry of funnel plots using Egger’s test, and *P* < 0.10 was considered a significant publication bias. Pooled estimates of the effect of recreational soccer on $\dot{V}{\text{O}}_{2\;\hbox{max} }$, using effect size (ES), were obtained using random effects models. Probabilistic magnitude-based inferences for meta-analysed effects were based on standardised thresholds for small, moderate and large changes (0.2, 0.6 and 1.2, respectively) derived from between-subject SDs for baseline fitness [32]. All statistical analyses were conducted using Comprehensive Meta-analysis software, version 2 (Biostat Inc., Englewood, NJ, USA). *P* < 0.05 was considered statistically significant.

## Results

### Study Selection

A total of 501 relevant studies was identified through database searching, and on the basis of their references an additional 14 articles were selected. After removal of duplicates, 319 studies remained. Based on a screening of the title and abstract, 273 articles were dismissed (196 excluded after title analysis; 77 excluded after abstract analysis). The full text of the 46 remaining papers was examined in more detail. Each study was read and coded for study characteristics, participant information, description of the training intervention and study outcomes. According to the eligibility criteria, 29 studies did not meet the inclusion criteria, while 17 studies that met the inclusion criteria were included in the systematic review and meta-analysis.

### Study Characteristics

All the studies that met the inclusion criteria were randomised controlled trials published in English between January 2009 and December 2014. The overall sample size was 380 participants, of whom 189 were female and 191 male. Eleven studies [1–4, 10–16] recruited male participants, five studies [33–37] recruited female participants and one study [9] recruited participants of both sexes. The age of the participants ranged from 19 to 76 years. Six studies investigated the effects of recreational soccer in healthy men [1, 4, 13–16], two in elderly men [1, 4] and five in healthy untrained women [33–37]. The remaining six studies investigated the effects of recreational soccer in patients with T2DM [3, 9], hypertension [2, 10, 11] and prostate cancer [12]. The training programmes lasted from 12 to 70 weeks, with specific durations of 12 [9–13, 15, 16, 37], 16 [4, 34, 35], 24 [3] 26 [2], 40 [33], 52 [1], 64 [14] and 70 weeks [36]. Small-sided games (3 vs. 3, 5 vs. 5 and 7 vs. 7) were the most frequent form of exercise during the interventions. One study [14] had a follow-up period of 52 weeks with training frequency reduced to 1.3 sessions per week. The most common training frequency was two to three sessions per week, with average subject participation of 1.3–2.8 training sessions per week. Soccer training sessions in each study lasted 40–60 min. Training intensity had average values of 78–84 % maximal heart rate (HR_max_), with the most common average intensity 82 % HR_max_. The fraction of total training time in the highest aerobic intensity zone, above 90 % HR_max_, varied from 12 to 30 %. Several of these studies used additional monitoring tools to describe locomotor activity and metabolic demands during training related to the effects on metabolic and musculoskeletal fitness [14], but these are not mentioned in the present manuscript dealing with effects on $\dot{V}{\text{O}}_{2\;\hbox{max} }$.

### Study Outcomes

**Fig. 2:**
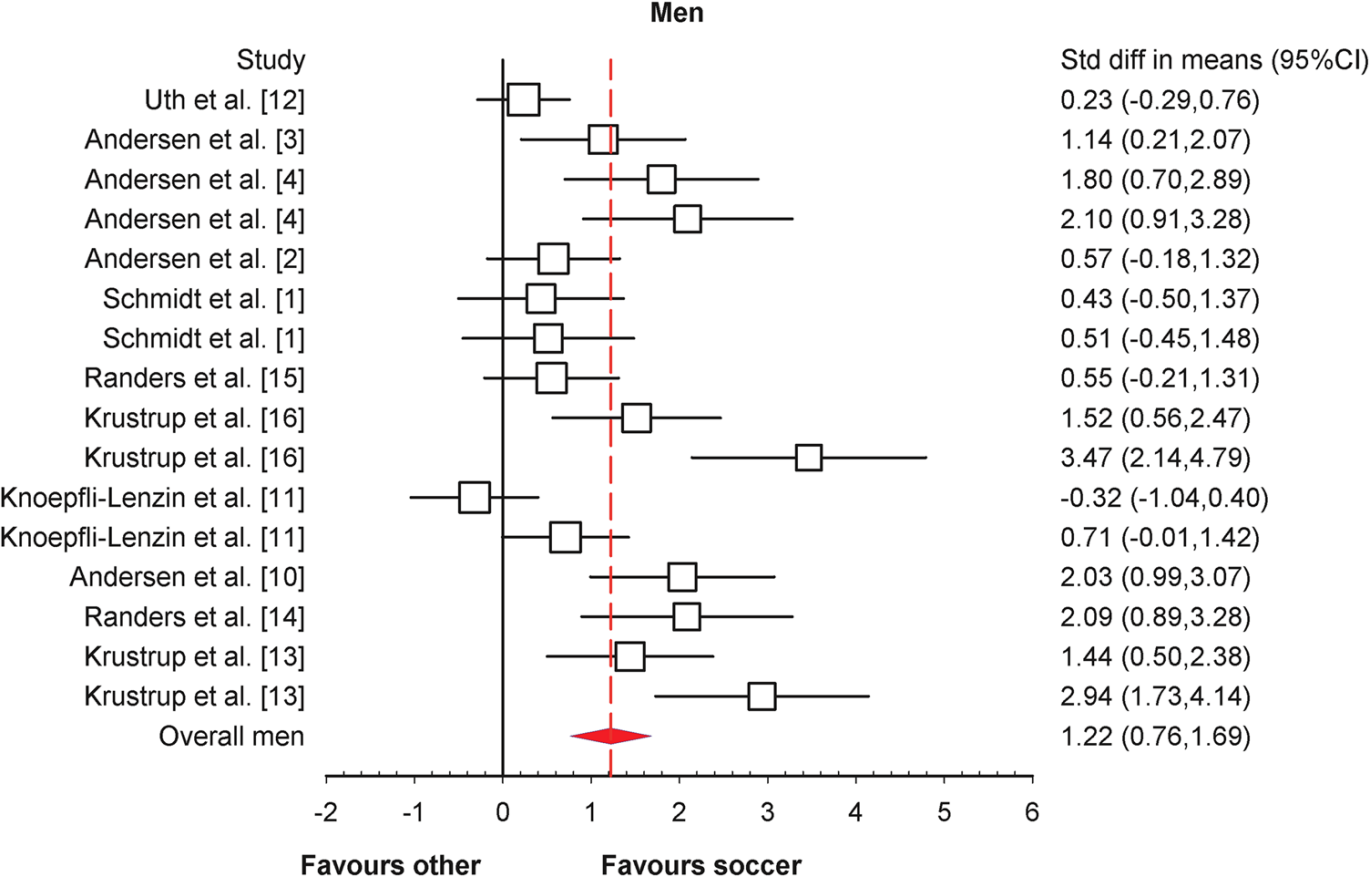
Forest plot of the effect sizes and 95 % confidence intervals (CIs) of the changes in maximal oxygen uptake after soccer training in men. *Std diff* standardised difference

**Fig. 3:**
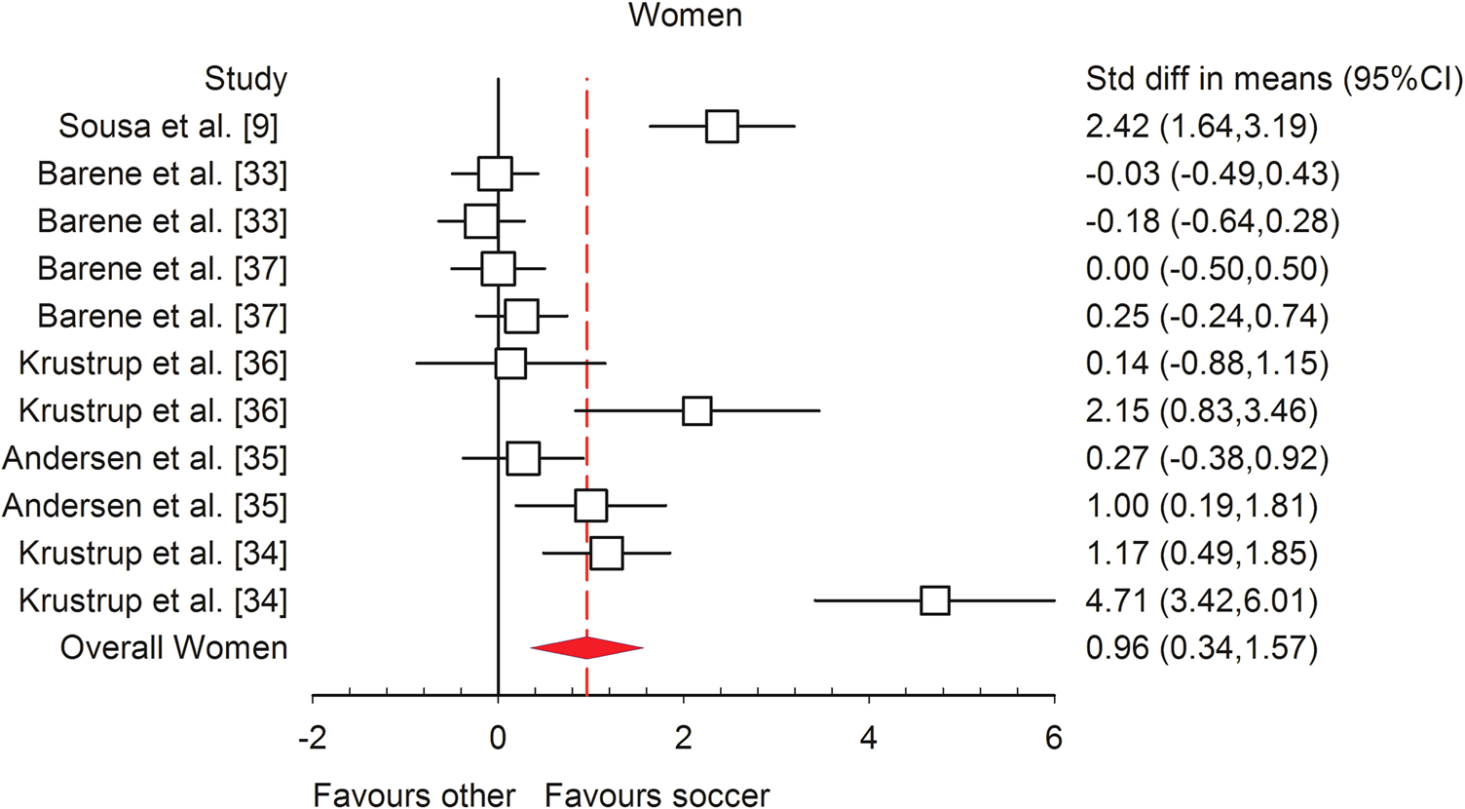
Forest plot of the effect sizes and 95 % confidence intervals (CIs) of the changes in maximal oxygen uptake in women. *Std diff* standardised difference

All of the studies that were included had enough data to calculate mean differences, ES and 95 % CIs. The statistically significant (*P* < 0.001) heterogeneity of the analysed studies was observed (*I*^2^ = 77.03 %), and for further analysis a random effect model was used. Differences in mean values showed that a recreational soccer training programme increased $\dot{V}{\text{O}}_{2\;\hbox{max} }$ by 3.51 mL/kg/min (95 % CI 3.07, 4.15; *P* < 0.001) in comparison with other training models. The meta-analysed effect on $\dot{V}{\text{O}}_{2\;\hbox{max} }$ of recreational soccer compared to controls was most likely largely beneficial (ES = 1.10; 95 % CI 0.73, 1.50; *P* < 0.001). When the results were analysed separately for men and women, the meta-analysed effect of recreational soccer on $\dot{V}{\text{O}}_{2\;\hbox{max} }$ was most likely largely beneficial in men (ES = 1.22; 95 % CI 0.76, 1.69; *I*^2^ = 76.55 %; Fig. 2) and most likely moderately beneficial in women (ES = 0.96; 95 % CI 0.34, 1.57; *I*^2^ = 90.72 %; Fig. 3) compared to all other investigated training regimens.

**Fig. 4:**
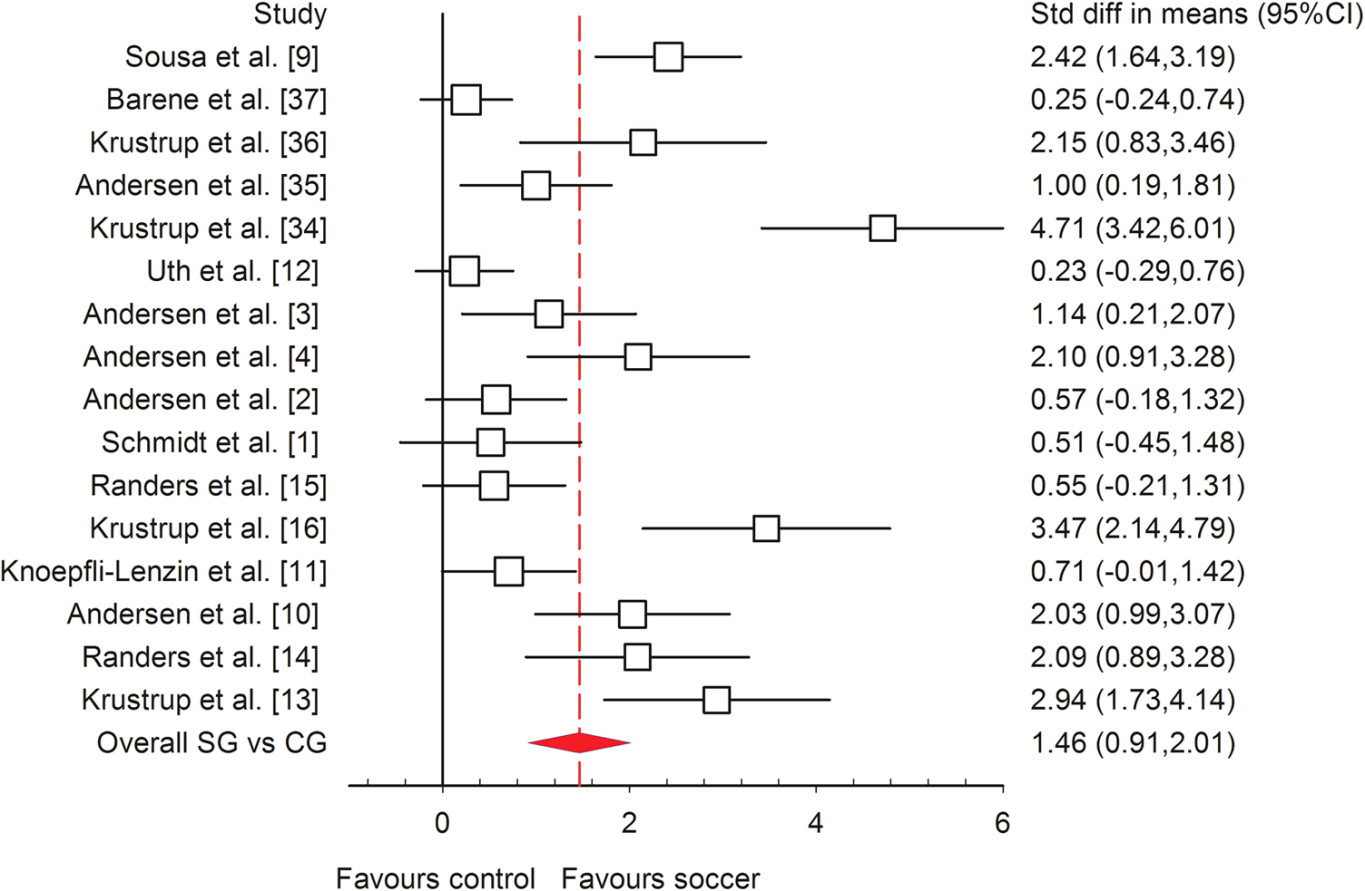
Forest plot of the effect sizes and 95 % confidence intervals (CIs) of the changes in maximal oxygen uptake. *CG* no-exercise group, *SG* soccer group, *Std diff* standardised difference

**Fig. 5:**
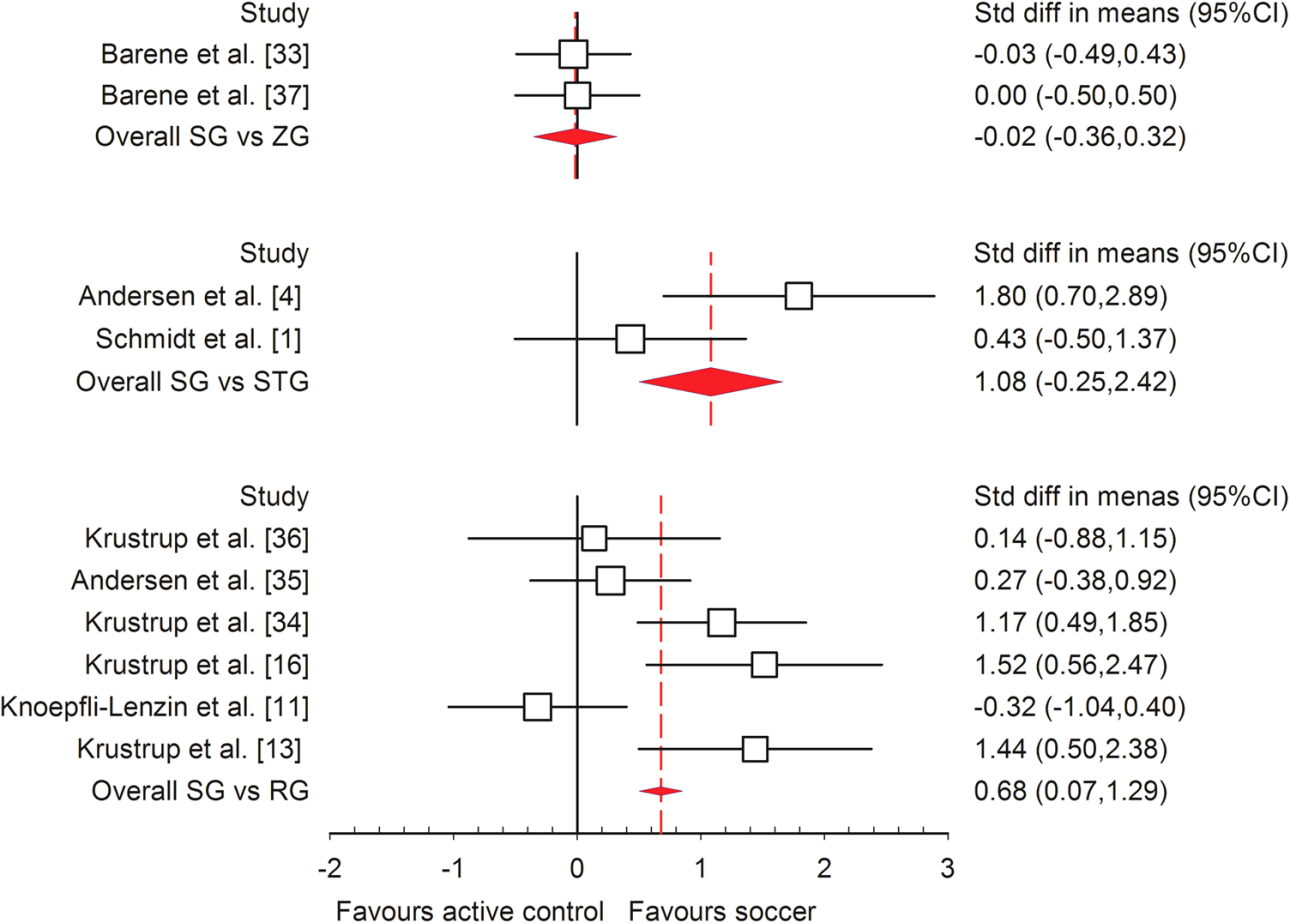
Forest plot of the effect sizes and 95 % confidence intervals (CIs) of the changes in maximal oxygen uptake by the type of control group. *RG* running group, *SG* soccer group, *Std diff* standardised difference, *STG* strength training group, *ZG* zumba group

The meta-analysed effects of recreational soccer on $\dot{V}{\text{O}}_{2\;\hbox{max} }$, when compared to different controls such as no exercise (Fig. 4), continuous running (Fig. 5) and strength training, were most likely largely beneficial (ES = 1.46; 95 % CI 0.91, 2.01; *I*^2^ = 88.35 %), most likely moderately beneficial (ES = 0.68; 95 % CI 0.06, 1.30; *I*^2^ = 69.13 %) and most likely moderately beneficial (ES = 1.08; 95 % CI −0.25, 2.42; *I*^2^ = 71.06 %), respectively. All studies investigating the influence of recreational soccer compared with a control group that did not have any kind of training programme showed ES favouring recreational soccer, ranging from 0.23 to 4.71. Ten of these studies [3, 4, 9, 10, 13, 14, 16, 34–36] showed a statistically significant effect (*P* < 0.05) for recreational soccer. The highest ES, most likely largely beneficial (4.71; 95 % CI 3.42, 6.01), was observed in healthy untrained women who had two sessions per week (average intensity 83 % HR_max_) and played 5 vs. 5, 7 vs. 7 and 9 vs. 9 matches over a period of 16 weeks [34]. The smallest ES was observed in a study [37] where the participants were healthy female hospital employees who performed two to three sessions per week lasting 60 min. In comparison with continuous running training, six studies [13, 16, 34–36] favoured recreational soccer, while only one study [11] showed that continuous running is better for $\dot{V}{\text{O}}_{2\;\hbox{max} }$ improvements, though the ES for this study was unclear (ES −0.32; 95 % CI −1.04, 0.40). Finally, when compared to strength training, both studies [1, 4] favoured recreational soccer, but only Andersen et al. [4] showed statistically significant differences (*P* < 0.001).

**Fig. 6:**
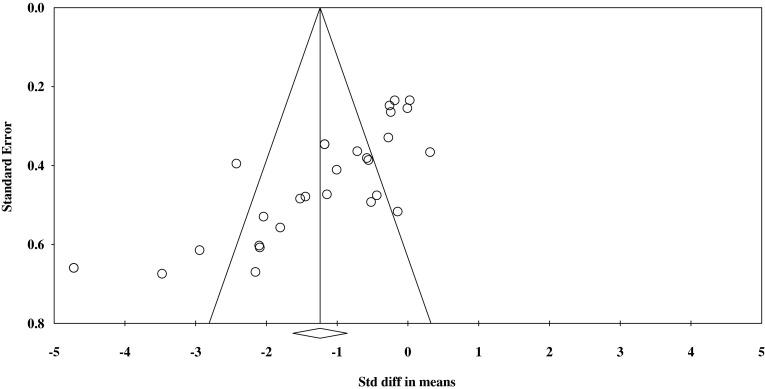
Funnel plot of standardised difference in mean effect size versus standard error. *Std diff* standardised difference

The Egger’s test was performed to provide statistical evidence of funnel plot asymmetry. The results indicated publication bias for the performed analysis (*P* < 0.10) (Fig. 6).

## Discussion

The main finding of this meta-analysis is that recreational soccer is effective for improving cardiorespiratory fitness and clearly produced better improvements in maximal aerobic capability than the other compared training programmes. The effect is likely to be largely beneficial in comparison with no exercise (ES = 1.46), while a moderate effect is observed compared with continuous endurance running (ES = 0.68) and strength training (ES = 1.08). Overall improvement equates to 3.51 mL/kg/min or a 10.3 % increase in $\dot{V}{\text{O}}_{2\;\hbox{max} }$ after short- to medium-term recreational soccer training. Those results are similar to those of previous meta-analyses [19, 20] that investigated the effects of HIIT versus no-exercise controls. Using similar inclusion criteria to the mentioned reviews, we observed a moderate effect on $\dot{V}{\text{O}}_{2\;\hbox{max} }$ improvements after recreational soccer training in comparison to continuous endurance running, while Gist et al. [20] and Milanović et al. [38] reported a trivial to small effect when comparing HIIT and continuous endurance running. However, we have not directly compared HIIT and recreational soccer so we cannot conclude that recreational soccer is better than HIIT, but our assumptions are based on results observed in similar meta-analyses.

Krustrup et al. [13] reported that recreational soccer and endurance running produce similar increases in $\dot{V}{\text{O}}_{2\;\hbox{max} }$ during the initial phase of training (first 4 weeks), namely 7 and 6 %, respectively. However, a further increase during the next 8 weeks was observed only in the recreational soccer group (6 %), while the stimulus of factors affecting $\dot{V}{\text{O}}_{2\;\hbox{max} }$ during the running training was not large enough for additional increases [39, 40]. One of the reasons for the bigger improvements in the soccer group is the marked and frequent change in exercise intensity when playing soccer, despite the fact that average heart rate was the same in the soccer and running groups. Usually during recreational soccer, ~20 % of the total training time comprises activities with intensity above 90 % HR_max_, compared with only 1 % for the continuous running group [13]. Similarly, previous meta-analysis [38] showed that HIIT is superior to continuous endurance running for $\dot{V}{\text{O}}_{2\;\hbox{max} }$ improvements. Thus, it is likely that high-intensity periods make recreational soccer training superior to continuous running in terms of producing improvements in $\dot{V}{\text{O}}_{2\;\hbox{max} }$ [13]. Unfortunately, no studies to date have directly compared recreational soccer and HIIT alone or a combination of high- and low-intensity training with the same training volume, so future investigations are warranted to compare the magnitude of improvements with these training methods.

Despite the fact that during recreational soccer heart rate is above 90 % HR_max_ for ~20 % of the time [13, 41], the rate of perceived exertion is lower than continuous running and much lower than interval training. Furthermore, psychological analysis showed that recreational soccer players did not express resistance to training and developed social interaction to a greater extent than the running group [13, 42]. Also, recreational soccer players were highly motivated to play during the study period as well as to continue playing after finishing the study [41, 42]. This observation was confirmed in follow-up studies of male participants [43, 44]. This is of major importance because lack of motivation is one of the key reasons for physical inactivity [6]. It seems that recreational soccer could be a promising type of physical activity for overcoming barriers such as cost efficiency, time efficiency, access to facilities and motivation. Furthermore, lack of time is the most common reason for inactivity and sedentary behaviour in people in both developing and developed countries [45]. In all the studies analysed in this meta-analysis, the training frequency for recreational soccer ranged from two to three sessions per week, but $\dot{V}{\text{O}}_{2\;\hbox{max} }$ improvement (~11 %) was similar, or in some cases superior, to training programmes following the American College of Sports Medicine (ACSM) recommendation of five training sessions per week. Accordingly, it seems as if recreational soccer is also time efficient. Randers et al. [14] observed that $\dot{V}{\text{O}}_{2\;\hbox{max} }$ increased markedly as a result of 1-h recreational soccer training sessions with a training frequency of two to three sessions per week over an initial 12 weeks, and that improvements in $\dot{V}{\text{O}}_{2\;\hbox{max} }$ and other markers of aerobic fitness could be maintained when the training frequency was decreased from 2.4 ± 0.5 sessions per week in the first 12 weeks to 0.9 ± 0.2 sessions per week for the last 28 weeks.

Positive effects of soccer training combined with a calorie-restricted diet on the $\dot{V}{\text{O}}_{2\;\hbox{max} }$ increment were found in female patients with T2DM [9]. No differences were found between female and male T2DM patients with regard to $\dot{V}{\text{O}}_{2\;\hbox{max} }$ improvement. A low level of aerobic fitness is a common characteristic in T2DM patients in comparison with non-diabetic subjects [46], and this was confirmed by the baseline values [9]. Soccer training organised as 3 vs. 3 or 7 vs. 7 over 12 weeks for 2 h per week improved $\dot{V}{\text{O}}_{2\;\hbox{max} }$ by 9.6 %. As recreational soccer combines aerobic high-intensity training, aerobic moderate-intensity training and resistance training [9], it results in intensity variation that increases $\dot{V}{\text{O}}_{2\;\hbox{max} }$ among T2DM patients. Higher aerobic capacity means that T2DM patients can spend more time being physically active and reduce their blood glucose level [47, 48]. Recreational soccer is also an appropriate type of physical activity for male T2DM patients and leads to an increase in $\dot{V}{\text{O}}_{2\;\hbox{max} }$ of ~10 % after only 12 weeks of training [3], similar to what has been reported in a meta-analysis of aerobic training in T2DM subjects [49]. The observed changes are important for T2DM patients because an increasing level of cardiorespiratory fitness of approximately 5 mL/kg/min is associated with a significant reduction in overall cardiovascular mortality of 39–70 % [50]. Aspenes et al. [51] found that 44.2 mL/kg/min represents a threshold below which the cardiovascular risk profile is unfavourable.

In many cases, recreational soccer is recognised as a male physical activity where females are still not included and do not actively participate. However, this meta-analysis confirmed that recreational soccer is an effective method for $\dot{V}{\text{O}}_{2\;\hbox{max} }$ improvements in women [9, 33–37, 52]. The study [34] with the highest change in $\dot{V}{\text{O}}_{2\;\hbox{max} }$ also had the highest change in ES (−4.72; 95 % CI −6.01, −3.42; *P* < 0.01) with a training intervention of four 12-min periods of small-sided games twice a week for 16 weeks and produced increases in $\dot{V}{\text{O}}_{2\;\hbox{max} }$ of 15.3 % in untrained premenopausal women. The $\dot{V}{\text{O}}_{2\;\hbox{max} }$ improvement in women occurs due to the relatively high-intensity exercise that soccer provides when played recreationally, irrespective of football skills and experience [34]. The average training intensity in the presented studies [34, 35] was 82–83 % HR_max_, with a large fraction of the training time in the highest aerobic training zone, i.e. above 90 % HR_max_. This emphasises that recreational soccer is intermittent in nature, involving a high number of intense actions and intense runs in multiple directions interspersed with low-intensity recovery periods [53], and can simulate interval training, which is proven to be an effective method for $\dot{V}{\text{O}}_{2\;\hbox{max} }$ improvement. Mean training frequency was 1.8 sessions per week, significantly lower than the 2.3 sessions considered to be the stimulus for elevating aerobic fitness in untrained men [13]. The reason for the higher $\dot{V}{\text{O}}_{2\;\hbox{max} }$ increment in women may be that baseline $\dot{V}{\text{O}}_{2\;\hbox{max} }$ was significantly lower in premenopausal women than in untrained men and the stimulus created by recreational soccer training was high enough to produce this improvement. The baseline level could define the percentage improvement in $\dot{V}{\text{O}}_{2\;\hbox{max} }$ because soccer training over 16 weeks increased $\dot{V}{\text{O}}_{2\;\hbox{max} }$ by only 8 % in subjects with relatively high maximal oxygen power [34, 54].

The lowest $\dot{V}{\text{O}}_{2\;\hbox{max} }$ percentage improvements (~3.4–4.6 %) were seen in hospital employees [33, 37] with similar training regimens over a 12-week intervention. Even though the training duration and frequencies in the study in question were similar to all the other meta-analysed studies (60 min; 2.3 times per week), the intensities were slightly lower, ranging from 78.3 to 78.6 %, than those found in the aforementioned studies [34, 35] with over 15 % $\dot{V}{\text{O}}_{2\;\hbox{max} }$ increase. In the study of hospital employees, there was a relatively high dropout rate in both exercise groups, i.e. zumba and soccer, and the intention-to-treat analyses carried out in this investigation seem to mask the large per-protocol effects. Actually, the improvement in $\dot{V}{\text{O}}_{2\;\hbox{max} }$ was as high as 10 % for the participants who trained more than two times per week over the 12-week period. The lower average improvement in $\dot{V}{\text{O}}_{2\;\hbox{max} }$ and the lower average attendance during the training intervention may also be related to the participant group and the setting. Working in a hospital can be stressful, with physiological fatigue occurring, especially if employees work more than 40 h per week [55]. Fatigue can disrupt $\dot{V}{\text{O}}_{2\;\hbox{max} }$ improvement by influencing the effectiveness of physiological adaptation [56]. The soccer training interventions involved after-work sessions, which may be the reason why $\dot{V}{\text{O}}_{2\;\hbox{max} }$ improvement was not at the same percentage level as in premenopausal [34, 35] and postmenopausal [9] women in previous studies.

Analysis of training interventions revealed a specific approach in terms of using small-sided games. One intervention [37] improved $\dot{V}{\text{O}}_{2\;\hbox{max} }$ by 4.6 % and consisted of training with one half-break of 5 min, while another [34] increased $\dot{V}{\text{O}}_{2\;\hbox{max} }$ by 15.3 % with three 2-min active breaks in a roughly identical 60-min recreational soccer protocol. Active breaks enhanced work capacity [57] by reducing blood lactate level and increasing aerobic energy yield [58]. Improvements in aerobic energy yield can be associated with a faster $\dot{V}{\text{O}}_{2\;\hbox{max} }$ kinetics during high-intensity bouts preceded by breaks [59]. Therefore, multiple active breaks have the ability to increase the effects of recreational soccer on the physiological adaptation process, resulting in improved $\dot{V}{\text{O}}_{2\;\hbox{max} }$ in women [37].

We propose several topics for future research to analyse recreational soccer in depth. Future studies should aim to identify the effects of different recreational soccer formats (3 vs. 3, 5 vs. 5, 6 vs. 6, 7 vs. 7, etc.) as well as combination of aforementioned formats on $\dot{V}{\text{O}}_{2\;\hbox{max} }$ and which, if any, is most suitable for different age categories and baseline fitness level. Also, the optimum weekly number and duration of training sessions is still unclear. All of the studies included in this meta-analysis used two to three training sessions per week lasting 40–60 min. However, these frequencies and durations are not in line with the ACSM recommendation. In addition, ACSM recommendations are largely based on continuous exercises and therefore may not be applicable to intermittent exercise-type games. Future studies should therefore investigate the optimum number and duration of training sessions for a wide range of subjects in respect of age, sex, health status and profession. This will help to produce prescriptions and recommendations for recreational soccer and its implementation in daily physical activity routines. Aside from the various benefits of recreational soccer, its effect in terms of injuries is still unclear, especially in adults and the elderly, although several studies provide evidence that the risk of injury during small-sided soccer training is only 10–20 % of the injury risk during 11 vs. 11 matches. A recent review by Oja et al. [60] has calculated the injury frequency during training studies with untrained healthy individuals across the lifespan and concluded that the injury risk is low during small-sided soccer training (1 per 500 h) as well as continuous running (1 per 700 h), whereas the injury risk was observed to be several-fold greater in a small-scale soccer training study with elderly men with prostate cancer undergoing anti-androgen treatment. Altogether, these findings support the use of the Football Fitness concept [61] with small-sided training sessions on small pitches in local football clubs, with proper warm-up including FIFA 11+ exercises and a main focus on training rather than matches.

## Conclusion

Recreational soccer produces large improvements in $\dot{V}{\text{O}}_{2\;\hbox{max} }$ compared to strength training and no exercise, regardless of the age, sex and health status of the participants. Also, recreational soccer is better than continuous endurance running, though the additional effect is moderate. Our meta-analysis provides evidence of the beneficial effects of recreational soccer on $\dot{V}{\text{O}}_{2\;\hbox{max} }$ in untrained men, homeless men, healthy premenopausal women and untrained hospital employees. The studies analysed confirmed that this type of activity is suitable for $\dot{V}{\text{O}}_{2\;\hbox{max} }$ improvement in untrained men and women with mild to moderate hypertension, T2DM patients, untrained elderly people and men with prostate cancer. Furthermore, recreational soccer is a highly motivating and social activity that appears to be very popular in significant parts of the population. It seems, therefore, as if recreational soccer has the potential to be implemented as a regular health-promoting physical activity, regardless of age, sex and health status. This kind of physical activity has the potential to enhance aerobic capacity, prevent and treat non-communicable diseases, and overcome lack of motivation, which is a key factor in physical (in)activity and immature levels of social habits. Recreational soccer is easy to organise, and there is a wide range of training types combining dynamic and intense movements with fast information processing. Just as interestingly, recreational soccer shows huge potential for transforming an untrained population into a physically active population. It is clear that recreational soccer organised as training sessions using small pitches and small-sided games, i.e. 3 vs. 3, 5 vs. 5, 7 vs. 7 or 9 vs. 9, positively affects $\dot{V}{\text{O}}_{2\;\hbox{max} }$.
